# Tooth number abnormality: from bench to bedside

**DOI:** 10.1038/s41368-022-00208-x

**Published:** 2023-01-06

**Authors:** Han Zhang, Xuyan Gong, Xiaoqiao Xu, Xiaogang Wang, Yao Sun

**Affiliations:** 1 Department of Implantology, Stomatological Hospital and Dental School of Tongji University, Shanghai Engineering Research Center of Tooth Restoration and Regeneration, Shanghai, China; 2 Key Laboratory of Big Data-Based Precision Medicine, School of Engineering Medicine, Beihang University, Beijing, China

**Keywords:** Disease genetics, Tissue engineering, Stem cells, Experimental organisms, Mesenchymal stem cells

## Abstract

Tooth number abnormality is one of the most common dental developmental diseases, which includes both tooth agenesis and supernumerary teeth. Tooth development is regulated by numerous developmental signals, such as the well-known Wnt, BMP, FGF, Shh and Eda pathways, which mediate the ongoing complex interactions between epithelium and mesenchyme. Abnormal expression of these crutial signalling during this process may eventually lead to the development of anomalies in tooth number; however, the underlying mechanisms remain elusive. In this review, we summarized the major process of tooth development, the latest progress of mechanism studies and newly reported clinical investigations of tooth number abnormality. In addition, potential treatment approaches for tooth number abnormality based on developmental biology are also discussed. This review not only provides a reference for the diagnosis and treatment of tooth number abnormality in clinical practice but also facilitates the translation of basic research to the clinical application.

## Introduction

Dental anomalies are characterised by abnormalities in the number, size, structure or shape of the teeth. Tooth number abnormality is a common human dental anomaly, including supernumerary teeth and tooth agenesis. Supernumerary teeth are defined as teeth that develop in addition to the regular number of teeth, including odontoma.^1^ Their prevalence varies from region to region and is between 0.2% and 5.3%.^2^ The maxillary anterior region is the most common site where a supernumerary tooth occurs. Tooth agenesis is the congenital absence of a tooth owing to the developmental arrest of the corresponding tooth germ,^3^ and its prevalence ranges from 1.6% to 6.9%.^4^ Congenital absence of mandibular incisors is the most common deciduous tooth agenesis. Tooth agenesis in the permanent dentition is common in the lateral incisors and second premolars. These two diseases cannot be treated with clinical intervention because their aetiology remains unclear.

The mouse is one of the most conventional animals used for exploring the intricate aetiology of tooth number abnormality, considering that the basic process of tooth development is similar among all jawed vertebrates. In the past decades, various mouse models have been used to study the causes of tooth number abnormality. The aetiologies of both supernumerary teeth and tooth agenesis are associated with mechanisms that govern tooth development, which have been extensively investigated from morphological, molecular and cellular perspectives. Development of tooth germ requires signalling centres and a series of events.^5–11^ Any minor change in signalling pathway molecules during the early stage of tooth development may lead to variations in tooth number. To understand the detailed underlying mechanisms, we summarised the developmental process and mouse models related to tooth number abnormality.

Considerable progress has been achieved in clinical studies on tooth number abnormality. Some clinical studies suggest that tooth number abnormality can contribute to the diagnosis of some important systemic diseases (such as related syndromes and tumours).^12–14^ Compared with the extended diagnostic significance, new advances in treatment are more noteworthy. Supernumerary teeth can provide postpartum stem cells,^15^ which can be used to relieve the symptoms of hepatic fibrosis and systemic lupus erythematosus.^16,17^ As a developmental biology-based treatment approach, genetic intervention has been used to treat tooth agenesis and supernumerary teeth in mouse models.^18–20^ Tissue engineering for whole-tooth regeneration is a promising therapeutic strategy for tooth agenesis and has been extensively investigated in mice, pigs and other animal models.^21,22^

In this review, we first summarised the basic research findings on tooth development, which can help to understand the mechanisms underlying tooth number abnormality. Subsequently, we summarised key clinical studies of tooth number abnormality, introduced the biological mechanisms study models of tooth number abnormality, and prospected novel treatment strategies.

## Process of tooth development

**Fig. 1:**
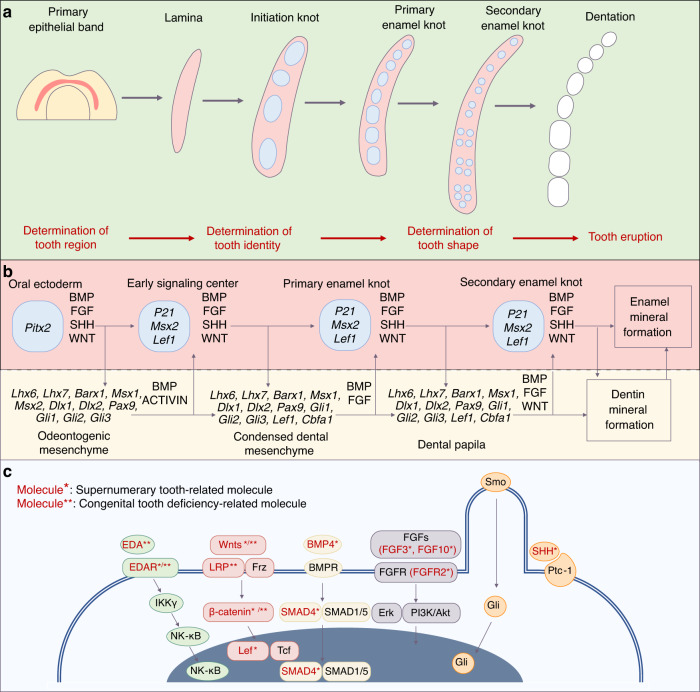
Tooth development process and summary of key odontogenic signal pathways. **a** The development process from primary epithelial band to dentation. **b** Molecules and signal pathways involved in tooth development. **c** Five key odontogenic signal pathways and related molecules involved in tooth number abnormality

Mammalian odontogenic processes are similar, and the signals involved are conserved.^23^ Tooth development in both humans and mice is regulated by several signalling centres involving multiple transcription factors and signalling pathways^24^ (Fig. 1). The primary epithelial band, which is characteristic of the initiation of tooth development, develops to dentition through many processes. Before tooth eruption, these processes are involved in determination of its region, identity and shape^25^ (Fig. 1).

### Tooth region determination

**Fig. 2:**
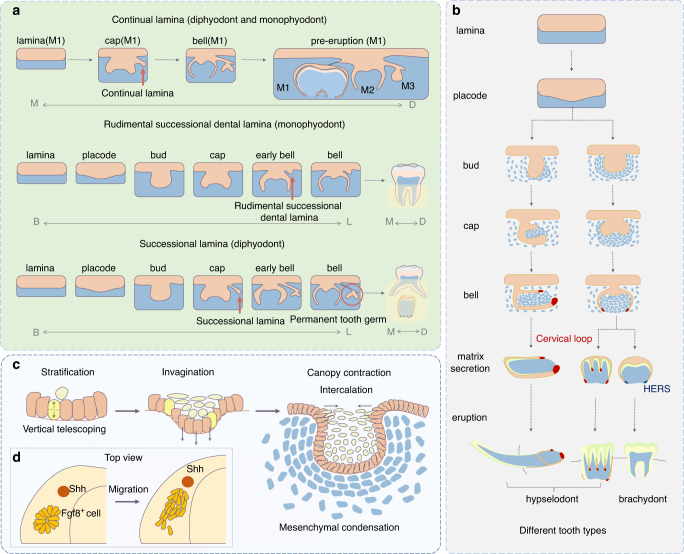
Schematic of tooth development process. **a** The continual lamina is present in diphyodonts and monophyodonts. The rudimental successional dental lamina cannot develop into a tooth under normal conditions, whereas the successional lamina forms the second dentition in diphyodonts. The dotted arrow indicates that a few parts of the tooth developmental process are omitted. B: buccal; L: lingual; M: mesial; D: distal; M1: first molar; M2: second molar; M3: third molar. **b** Different tooth types are classified according to the clinical crown-to-root ratio and the self-renewal ability after eruption. **c** Cell behaviour during early tooth development. Yellow cells form the suprabasal cells (light yellow) via asymmetric division; orange cells undergo vertical expansion. Grey–blue mesenchymal cells condense around the epithelium. **d** Top view of the migration of *Fgf8*^*+*^ epithelium in the lower jaw

The human tooth germ originates from ectoderm based on the interaction between the original oral epithelium and ectomesenchyme derived from the neural crest. At approximately 4 weeks of gestation, the epithelium begins assuming odontogenic (tooth-forming) capacity and proliferates to form a continuous U-shaped band called the primary epithelial band. The formation of this band signifies the initiation of tooth development. As the embryo develops, the band extends into the underlying mesenchyme and gradually forms two branches: the dental and vestibular laminae. The dental lamina is the source of subsequent activity and differentiation relative to tooth development, whose expansion may lead to the development of supernumerary teeth.^26^ It can be classified as continual and successional laminae. The continual lamina horizontally forms from the posterior extension of the dental lamina,^27^ whereas the successional lamina vertically forms from the lingual side of the dental lamina. The successional lamina eventually forms a second dentition (permanent dentition) by disconnecting from the oral epithelium through apoptosis and epithelial-to-mesenchymal transition^28^ (Fig. 4a). A few monophyodonts, such as mice, possess the rudimental successional dental lamina, which is similar to the human successional lamina but disappears after birth owing to cell proliferation cessation^29^ and connective tissue and capillary invasion in the dental stalk^30^ (Fig. 2a).

The formation of lamina from the primary epithelial band is a complex process, which is accompanied by the expression of many odontogenic transcription factors. *Pitx2*0, *Foxi3*, *Dlx2*, *Lef1* and *p63* are specifically expressed in the dental lamina.^10,11^ Early tooth development relies on the proper expression of these factors. *Pitx2* is among the earliest markers of tooth development, such as *Lef1*, and interacts with β-catenin.^31^ *Pitx2*, *Lef1* and *Sox2* can participate in the transcriptional mechanism to regulate the steady state of dental epithelial stem cells (EpSCs)^32^ and formation of dental signalling centres.^33^ Sox2 can inhibit the transcriptional activity of Pitx2 to repress the activation of *Lef1* promoter by Pitx2–Lef1.^33,34^ *Pitx2* deficiency, in turn, delays the invagination of both dental epithelium and vestibular lamina, with reduced *Shh* expression.^33^ *p63* is crucial for the formation of the dental lamina. A gene regulatory network dominated by *p63*, which can regulate the adhesion, polarity and migration of odontogenic cells, may exist during the determination period of the dental-forming region.^35^ Deficiencies of these transcription factors (*Pitx2*, *Lef1* and *P63*) inhibit early tooth development, consequently leading to missing teeth.^33,36–40^

The developmental stage subsequent to the laminal stage is the placode stage (Fig. 2b). The formation of all epidermal appendages, including teeth, begins with the placode stage, and the formation of placodes signifies initiation of the formation of individual tooth.^10^ Cellular behaviours during early tooth development include cell migration, intercalation and condensation.^6–9^ Abnormalities in these behaviours can affect the number of teeth. From the top view, a cluster of *Fgf8*^+^ dental epithelial cells appear to be arranged in a rosette formation. As the embryo develops, these cells migrate to the mesial Shh signalling centre (possibly the initiation knot [IK]) and form a placode.^7^ From the sectional view, complex cellular behaviour is observed. Epithelial monolayer columnar cells migrate upward relative to their adjacent central cells, with their apex protruding toward the centre. This process is known as vertical telescoping.^8^ Simultaneously, the FGF signalling-dependent division of perpendicular cells produces suprabasal cells to stratify the epithelium. Subsequently, Shh signalling triggers the rearrangement of cells in the tissue, driving an epithelial invagination^9^ (Fig. 2c). However, epithelial evagination owing to the perturbation of Wnt signalling results in the formation of supernumerary teeth.^41^

### Tooth identity determination

The bud stage subsequent to the placode stage is important for the determination of tooth identity. As the placode develops, suprabasal cells located at the canopy horizontally intercalate and centripetally migrate, pushing the epithelium to embed in the mesenchyme while narrowing the tooth germ neck. This process, known as canopy contraction, contributes to the gradual transformation of the epithelium into a bud shape.^42^ Subsequently, mesenchymal cells respond to FGF8 and SEMA3F secreted by the epithelium to condensate around the bud-shaped epithelium with increasing collagen VI expression. This process induces the mesenchyme to express odontogenic transcription factors (Pax9 and Msx1) by inhibiting the mechanical signalling molecule Rho A^43–45^ (Fig. 2c). During this process, the odontogenesis potential and tooth identity information are transferred from the epithelium to mesenchyme, with mesenchymal condensation and expression of specific odontogenic molecules.^46–50^

Recently, it was found that a few epithelial cells condensate to form a signalling centre known as IK, which sends signals to neighbouring cells, thereby inducing proliferation to complete the placode-to-bud transition.^51^ IK is silenced via apoptosis at the bud stage.^5,52^ IK with specific Eda and NFkB signalling was originally observed in the incisors, and its size affects the incisor germ size.^53^ The molar IK has high expression of Wnt and Shh, and interference of IK formation via physical ablation or Wnt signal confusion can prevent the development of the tooth germ.^5^

### Tooth shape determination

After the determination of tooth identity, different types of tooth germ begin to show morphological differences. Different types of teeth form their unique shapes during this process, which is difficult to achieve in tissue-engineered tooth regeneration. Compared with the previous two stages, the possibility of developing tooth number abnormalities is reduced; however, tooth agenesis may occur owing to the arrest of tooth germ development in the cap or bell stage.^32,54^

The process of tooth shape determination includes the bud, cap and bell stages (Fig. 2b). The morphological features of teeth are predominantly controlled by signal centres known as enamel knots (EKs) during tooth shape determination. Single-cusp teeth (e.g., incisors) only possess primary EKs (pEKs), whereas poly-cusp teeth (e.g., molars) possess two signal centres, namely, pEKs and secondary EKs (sEKs). In the late bud stage of the molars, a group of apoptotic cells is concentrated at the tip of the tooth bud, with the expression of *p21.*^55^ *P21*^+^ cells, which are insensitive to proliferative signals, congregate in the basal layer of the epithelial centre, forming a pEK.^56^ Various signals regulate pEK formation during the late bud stage. Sufu inhibition in the mesenchyme or Gpr177 deletion in the epithelium can lead to the failed formation of a functional pEK, which further results in developmental retardation or arrest of the tooth germ.^54,57^ Subsequently, the molar tooth germ forms a new signal centre (sEK) during the cap-to-bell transition. The molar anterior buccal sEK, the first sEK, is derived from the pEK.^58^ and consists of non-proliferating cells consistent with the pEK, whereas the second sEK established through *de novo* signalling contains dividing cells.^59^ All sEKs are located in the inner enamel epithelial region where the tooth cusps initiate.^60^

## Clinical studies of tooth number abnormalities

Tooth number abnormalities are common dental disease in humans. They can occur alone or accompanied by other diseases. For non-syndromic tooth number abnormalities, the general treatment could achieve satisfactory results. Whereas syndromic tooth number abnormalities require specific treatment strategies tailored to an individual’s complications or related symptoms.

### Clinical feature and treatment strategies of supernumerary tooth

The occurrence features of supernumerary teeth lacks a uniform description. Study findings vary from region to region, possibly owing to sample differences.^61^ For instance, the incidence of supernumerary teeth is ~0.05% in Japanese children and ~3.2% in Mexicans.^62,63^ With respect to sex, several studies have reported that men have a higher risk of developing supernumerary teeth.^61,64,65^ Additionally, the preferred sites for the occurrence of supernumerary teeth may be sex-related.^66^ However, a few studies have reported that the incidence of supernumerary teeth does not significantly differ between men and women.^67,68^

Treatment strategies for supernumerary teeth are improved constantly. The general treatment option for non-syndromic supernumerary teeth is extraction. Additionally, surgical intervention is required in patients with complications or related syndromes.^69–71^ However, if supernumerary teeth are functionally and aesthetically significant and there is a loss of permanent teeth in the dentition, supernumerary teeth in the dentition can be considered replacement teeth.^72^

### Clinical feature and treatment strategies of tooth agenesis

Tooth agenesis can occur as an isolated disease (non-syndromic tooth agenesis) or can be associated with a syndrome (syndromic tooth agenesis). Several syndromes, including Down syndrome, ectodermal dysplasia and labio-palatal cleft have been associated with severe or moderate tooth agenesis.^3^ Varied missing teeth sites are found in different syndromes. The common missing tooth site in patients with Down syndrome is the second premolar of the left lower jaw, whereas patients with a cleft lip are more likely to have missing superior incisors.^73,74^

Tooth agenesis can be definitively diagnosed via imaging; however, the treatment options cannot be generalised because tooth agenesis is often associated with alterations and deformities in the tooth structure, delayed eruption and tooth displacement. For instance, patients with tooth agenesis caused by Wnt10B mutation may also have microdontia and taurodontism.^75^ In patients with tooth agenesis caused by Pax9 mutations, the middle incisors in the upper jaw are susceptible to microdontia,^76^ and regional odontodysplasia may also occur.^77^ In addition, tooth agenesis may affect the oral arch length, jaw position and craniofacial morphological features.^78,79^ Therefore, imaging is necessary for early diagnosis, prompt intervention^80^ and multidisciplinary treatment to maintain the aesthetic and functional features of teeth.

## Pathogenetic mechanisms of tooth number abnormality

The different occurrence features and related symptoms hint at the complex mechanisms of tooth number abnormality. However, the exact pathogenesis is still unclear. To explore it, many related mouse models have been constructed in laboratories (Table 1, Table 2). Based on the research progress, the appropriate stimulation of signalling pathways are the keys to determining the number of teeth. A few minor disturbances can inhibit development of the tooth germ or lead to the formation of supernumerary teeth.^13,27,81^

**Table 1:** Mouse models of supernumerary tooth

Pathway	Mouse model	Phenotype	Reference
Wnt	*R-spondin2*^*−/−*^	Mesiodistal supernumerary teeth in the diastema region	^96^
	*K14-Cre*^8*Brn*^*; Apc*^*cko/cko*^	Numerous supernumerary teeth surrounded the principal teeth	^88^
	*Fgf8*^*CreER*^*; Ctnnb1*^*Δex3fl/+*^	Numerous supernumerary teeth surrounded the principal teeth	^41^
	*K14-Cre Ctnnb1*^*(Ex3) fl/+*^	Numerous supernumerary teeth surrounded the principal teeth	^89,90^
	*Wise*^*−/−*^	Supernumerary teeth in the incisor, molar, and diastema regions	^93,94^
	*Lrp4*^*−/−*^	Supernumerary teeth in the incisor and molar regions	^92^
	*Wise*^*−/−*^*, Lrp5*^*−/−*^	Supernumerary teeth in the incisor and diastema regions	^93^
	*Wise*^*−/−*^*, Lrp5*^*+/−*^	Supernumerary teeth in the incisor and diastema regions	^93^
	*Wise*^*−/−*^*, Lrp5*^*+/−*,^ *Lrp6*^*+/−*^	Mesiodistal supernumerary teeth in the diastema regions	^93^
	*Wnt10a*^*−/−*^	Mesiodistal supernumerary teeth (M4) in the molar region	^97^
FGF	*Spry2*^*−/−*^	Mesiodistal supernumerary teeth in the diastema region	^112,113^
	*Spry2*^*+/−*^*Spry4*^*−/−*^	Supernumerary teeth in the incisor region	^184^
	*Spry4*^*−/−*^	Mesiodistal supernumerary teeth in the diastema region	^112^
	*K14-Cre; R26R-fgf8*	Buccolingual supernumerary teeth in the incisor and molar regions	^185^
Shh	*Gas1*^*−/−*^	Mesiodistal supernumerary teeth in the diastema region	^103,186^
	*Wnt1-Cre; Polaris*^*flox/flox*^	Mesiodistal supernumerary teeth in the diastema region	^186^
	PCS1-MRCS1^Δ/Δ^	Mesiodistal supernumerary teeth in the diastema region	^101^
	ΔMRCS1/ΔMRCS1	Mesiodistal supernumerary teeth in the diastema region	^102^
	ΔMRCS1*/Shh* KO	Mesiodistal supernumerary teeth in the diastema region	^102^
	Injection of 5E1 (at E12) *	Mesiodistal supernumerary teeth in the diastema region	^105^
	Injection of 5E1 (at E14) *	Buccolingual supernumerary teeth in the incisor and molar regions	^105^
EDA	*K14-Eda-A1*	Mesiodistal supernumerary teeth in the diastema region	^187^
	*K14-Eda*	Mesiodistal supernumerary teeth in the diastema region	^118,187^
	*K14-Eda; Fgf20*^*−/−*^	Mesiodistal supernumerary teeth in the diastema region	^120^
	*K14-Edar*	Mesiodistal supernumerary teeth in the diastema region (sometimes missing third teeth)	^118^
	*B6CBACa-A*^*w-J*^*/A-Eda*^*Ta*^*/0*,	Mesiodistal supernumerary teeth in the diastema region	^188^
	*Heterozygous Tabby*	Mesiodistal supernumerary teeth in the diastema region (sometimes missing third teeth)	^189^
	*Heterozygous Eda* ^*Ta/+*^	Mesiodistal supernumerary teeth in the diastema region	^189,190^
Others	*Rsk2*^*–/Y*^	Mesiodistal supernumerary teeth in the diastema region	^191^
	*Tg737*^*orpk*^ *mutant*	Mesiodistal supernumerary teeth in the diastema region	^186,192^
	*K14-Cre; Fam20B*^*flox/flox*^	Buccolingual supernumerary teeth in the incisor region	^115,116^
	Cebpb^−/−^; Runx2^+/−^	Buccolingual supernumerary teeth in the incisor region	^193^
	*Sey/Sey*	Supernumerary teeth in the upper incisor region	^194^
	*Di*	Buccolingual supernumerary teeth in the incisor region	^195^
	Organ culture of mesenchyme-trimmed germ	Extra incisors develop in wild-type explants when most of the surrounding mesenchyme is removed before culture	^94^
	*Osr2*^*−/−*^	Buccolingual supernumerary teeth in the molar region	^108,109^
	Epiprofin^−/−^	Numerous supernumerary teeth in the incisor and molar regions	^196^
	*Pitx2-Cre; Irf6* ^*F/F*^	Supernumerary teeth in the incisor and diastema regions	^197^

*5E1: an IgG1 monoclonal antibody against Shh protein

**Table 2:** Mouse models of congenital tooth deficiency

Pathway	Mouse model	Phenotype	Reference
Wnt	*Wnt7b-expressing*	Tooth germ development arrested at the bud stage	^99^
	*Fgf8CreER; Ctnnb1*^*f/f*^	Tooth germ development arrested at the placode–bud transition stage	^41^
	*Osr2-cre*^*KI*^*; Ctnnb1*^*ex3f*^	Tooth germ development arrests at the cap stage	^133^
	*Osr2-IresCre; Catnb*^*f/f*^	Tooth germ development arrested at the bud stage	^150^
	*Lef1*^*−/−*^	Tooth germ development arrested at the bud stage	^37–40^
	*Dkk1-expressing*	Molar tooth germ development arrested at the lamina–early bud stage Incisor tooth germ development arrested at the placode stage	^89^
	*K14-Dkk1 transgenic mice*	Molar tooth germ development arrested at the lamina–early bud stage	^134^
	*Wnt1-Cre; Rspo3*^*fl/−*^*; Rspo2*^*+/+*^	Tooth germ development arrested at the bud stage	^135^
	*K14-Cre; Apc*^*cko/cko*^	Incisor agenesis	^198^
Shh	*Gli2*^*−/−*^*; Gli3*^*+/−*^	Tooth germ development arrested at the placode stage	^138^
	*Gas1*^*−/−*^*; Shh*^*+/−*^	Premaxillary incisor agenesis	^104^
	*K14-Shh*	Tooth germ development arrested at the bud stage	^199^
BMP	*Wnt1Cre; Bmp4*^*f/f*^	Tooth germ development arrested at the bud stage	^110,141^
	*Osr2*^*−/−*^*Wnt1Cre; Bmp4*^*f/f*^	Supernumerary tooth germ development arrested at the placode–bud transition stage	^141^
	*Bmp4*^*ncko/ncko*^ *Inhba*^*−/−*^	Tooth germ development arrested at the bud stage	^110^
	*Inhba*^*−/−*^	Tooth germ development arrest at the bud stage	^110^
	*K14-Cre43; Bmpr1a*^*cl/cl*^	Tooth germ development arrested at the bud stage	^140^
	*Wnt1-Cre; Smad4*^*fl/fl*^	Tooth germ development arrested at the lamina stage	^142^
	*K14Cre; pMes-Nog*	Tooth germ development arrested at the lamina/early–bud stage	^139^
FGF	*Fgf3*^*−/−*^*; Fgf10*^*−/−*^	Tooth germ development arrested prior to bud stage	^154^
	*Fgfr2(IIIb)*^*−/−*^	Tooth germ development arrest at the bud stage	^152^
	*K14-Cre; Fgfr2*^*fl/fl*^	Retarded tooth formation	^153^
Eda	*K14–Edar (Intermediate)*	Third molar agenesis	^200^
	*Pax9*^*+/*^^*−*^*Eda*^*−/−*^	Incisor and third molar agenesis	^162^
TF	*P63*^*−/−*^	Tooth germ development arrested at the dental lamina stage	^36^
	*Krt14-Cre; Pitx2*^*flox/flox*^	Tooth germ development arrested at the bud stage	^33^
	*Msx1*^*−*^^*/−*^	Tooth germ development arrested at the bud stage	^143,144^
	*Msx1*^*−/*−^ *Msx2*^*−/−*^	Tooth germ development arrested at the dental lamina–early bud stage	^145^
	*PAX9*^*−/−*^	Tooth germ development arrested at the bud stage	^159^
	*Pax9*^*lacZ/lacZ*^	Tooth germ development arrested at the bud stage	^163^
	*Pax9*^*neo/lacZ*^	Tooth germ development arrested at the bell stage	^163^
	*Pitx2Cre; Sox2*^*F/F*^	Tooth germ development halted at the late bud-bell stage	^32^
Others	*Gpr177* ^*K14cre*^*; Osr2*^*−/−*^	Supernumerary tooth germ development arrested at the lamina/early bud stage	^54^
	*Gpr177* ^*K14cre*^	Tooth germ development arrested at the early cap stage	^54^
	*Wnt1-Cre; Alk5*^*fl/fl*^	Tooth germ development delayed	^201^
	*K14Cre; Ctnna1*^*cKO*^	Tooth germ development arrested at the bud stage	^202^
	*EL/Sea*	Third molar agenesis	^203^
	*K14-follistatin*^*−/+*^	Third molar agenesis	^204^
	*K5-GR*	Second and third molar agenesis	^205^
	*Pitx2-Cre; Irf6* ^*F/F*^	Third molar agenesis	^197^

*TF* transcription factor

### Signalling pathways related to supernumerary tooth

In human, the aetiology of supernumerary teeth is multifactorial and involves both genetic and environmental factors.^82^ Although the genetic propensity of supernumerary teeth is not consistent with a simple Mendelian pattern, they are more common in the relatives of patients than in the general population.^83^ To date, multiple genes have been identified in supernumerary teeth cases, such as *APC*, *RUNX2, FAM20A* etc..^84–86^ And related mouse models have been employed to explore the pathogenesis **(**Table 1**)**.

#### Wnt signalling pathway

The Wnt signalling pathway genes play important roles in the supernumerary tooth. Among them, *APC* is one of the most famous pathogenicity genes in human patients.^85^ Its product inhibits the Wnt signalling pathway by down-regulating β-catenin protein.^87^ And *APC* mutation results in the classical Wnt signalling pathway anomaly, thereby forming supernumerary tooth.^84^

**Fig. 3:**
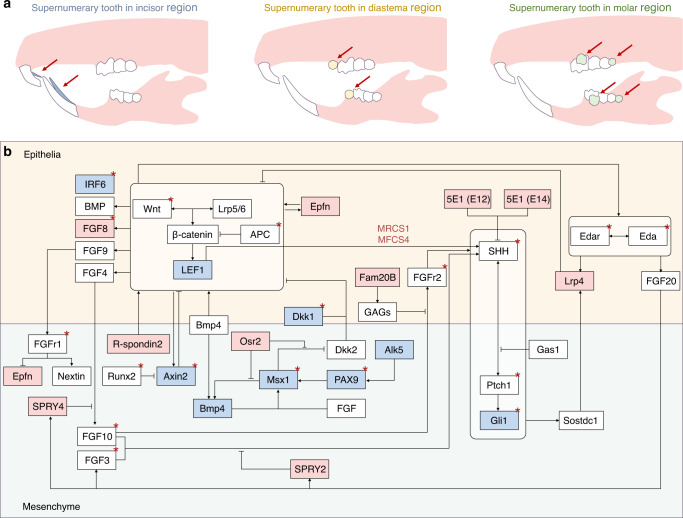
Schematic of location and genes regarding tooth number abnormality. **a** Schematic of supernumerary teeth located in different regions. **b** Summary of genes related to tooth number abnormality. Red represents genes associated with extra teeth; blue represents genes associated with inhibition of tooth development; Red * represents genes reported in humans

Investigations also support the activation of Wnt signalling and the formation of extra teeth. In the dental epithelium, overactivation of epithelial Wnt/β-catenin signalling owing to stabilisation of β-catenin or ablation of the Wnt inhibitor Apc leads to the occurrence of extra teeth.^88^ The epithelial overactivation of β-catenin produces a large domed evagination of the epithelium with mesenchymal condensation,^41^ thus expanding the expression regions of Wnt10b, Lef1, Bmp4, Msx1 and Msx2 and eventually leading to the formation of extra teeth.^89^ In particular, multiple small teeth surround a larger incisor, and the molar region usually forms dozens of tapered teeth.^90^ Besides, Wise (SOSTDC1, ectodin and USAG1) serves as an inhibitor of Lrp5- and Lrp6-dependent Wnt signalling and contributes to signal conduction between epithelium and mesenchyme, thus limiting the number of teeth.^91,92^ Its mutation can lead to supernumerary teeth in mouse^93^ (Fig. 3). And the extra incisor phenotype of Sostdc1-deficient mice can be replicated by activating Wnt signalling via reducing the mesenchymal tissue around the incisor tooth germ.^94^

However, function of Wnt signalling is complex during supernumerary tooth formation. On the one hand differences in the location of overactivated Wnt signalling lead to contradictory phenotypes. Overactivation of mesenchymal Wnt/β-catenin signalling in vitro inhibits the formation of M2 and M3.^95^ On the other hand, downregulation of Wnt signalling may also lead to supernumerary tooth. Mutation in R-spondin2, a Wnt signalling activator, can lead to the formation of an extra tooth in diastema with significant reduction of Wnt signalling activity; however, no significant abnormalities occur in molars or incisors.^96^ In addition, the role of Wnt signalling in supernumerary tooth is not completely identical in humans or mice. Some mice with *Wnt10a* mutation (~50%) have the fourth molar, whereas it has not been reported in patients with *Wnt10A* mutation.^97^

#### SHH signalling pathway

Shh signalling pathway is an essential signalling pathway regulated tooth number. Shh, Wnt and their interactions are significant factors regulating the boundaries of the odontogenic region. And Shh signalling pathway is both the downstream signal and negative feedback regulator of Wnt signalling during tooth development^98,99^ According to a clinical study, Shh can be a molecular biomarker in children with supernumerary teeth due to its higher expression level in patients with supernumerary teeth.^100^ And modulating SHH signals can lead to the formation of extra teeth in mice. MRCS1 and MFCS4 are enhancers that regulate Shh expression in the mouse tooth epithelium.^101^ MRCS1 can interact with Lef1/Tcfs and is regulated via Wnt/β-catenin signalling. The loss of these enhancers can result in the formation of an extra tooth in front of M1.^102^ Gas1, a protein located in the mesenchyme during tooth development, can regulate the number of teeth. It can limit Wnt and FGF signals in the odontogenic epithelium by regulating Shh signal transduction.^103,104^

Furthermore, a lingual supernumerary tooth model can be constructed by modulating Shh signalling during the special stage of tooth development. 5E1 is a monoclonal antibody against the Shh protein. Injecting 5E1 at E12 results in the formation of a supernumerary tooth in the diastema, whereas injecting it after 2 days (E14) results in the formation of lingual teeth in the incisor and molar regions.^105^ This phenomenon indicates that odontogenic regulation of Shh signalling is time-specific. Supernumerary teeth located on the lingual side of normal teeth occur more commonly than those on the buccal side, which may be associated with abnormalities in the successional lamina.

#### BMP signalling pathway

BMP signalling pathway is an essential tooth development-related signalling that also involves the formation of tooth number abnormality. The related genes including *BMPR1A, BMPR2, BMP6, BMP2*, and *SMAD6* have been identified in mesiodens patients. According to gene co-occurrence network analysis BMP, SHH, and WNT signalling pathways together form a genomic alterations network of supernumerary tooth.^106^

BMP4 is one the most crucial molecules in BMP related genes in mouse tooth germs. It constitutes a signalling axis with Msx1 to regulate tooth development. BMP4 is initially expressed in the epithelium but induces the transcription factors MSX1 and MSX2 in the mesenchyme. Subsequently, the expression of mesenchymal BMP4 increases in an MSX1-dependent manner, and BMP4 returns to the epithelium, inducing the expression of P21.^89^ The Msx1–Bmp4 axis inhibits Sfrp2 and Dkk2. And Osr2 can inhibit this axis, which is a negative regulator of dental signalling, predominantly in the mesenchyme.^107^ In *Osr2*^*−/−*^ mice, with the increased expression of odontogenesis-related signalling molecules (such as BMP4), extra teeth form at the lingual side of molars, which resemble the second row of teeth in non-mammals.^108,109^ Moreover, the loss of Osr2 can rescue the arrested molar germ development in *Inhb*^*−/−*^ mice.^110^

#### FGF signalling pathway

FGF signalling pathway connects with the formation of supernumerary tooth. Controlling some vital FGF-related molecules directly is a typical method to construct a supernumerary tooth model. FGF8 can activate odontogenesis in the diastema region.^111^ If epithelial FGF8 is ectopically activated directly, genes related to tooth development, such as Pitx2, Sox2, Lef-1, p38 and Erk1/2, are expressed in the incisor lingual epithelium, thereby activating the tooth-forming ability. And loss function of Sprouty (Spry), a negative feedback regulator of FGF also can lead to the formation of supernumerary tooth.^112,113^

Indirectly interfering with FGF signalling pathways is another pathogenic mechanism of supernumerary tooth. The molecular mechanism of Cleidocranial dysplasia (CCD) related to *Runx2* haploinsufficiency involves the activated FGF signalling by freely excessive unbound Twist1.^114^ Besides, FAM20B is a xylose kinase that is required for glycosaminoglycan (GAG) assembly. Additionally, during the initial stages of tooth development, GAG can limit FGFR2b signalling to regulate the cell fate of the incisor lamina and maintain the balance between the proliferation and differentiation of Sox2^+^ cells.^115^ Therefore, after the knockout of Fam20B in the epithelium, supernumerary teeth can appear on the lingual side of the incisor region.^116^

#### EDA signalling pathway

Abnormalities Eda signalling pathways also activate the odontogenesis potential. It is identified as morphogenic signalling regulating the formation of tooth including shape, size and number according some developmental researches.^117,118^ Supernumerary tooth models can be constructed using *K14-Eda* mice,^118,119^ and inactivation of Fgf20 increases the probability of developing extra teeth in *K14-Eda* mice.^120^ Besides the modulation of EDA signalling pathway on dental formula is also explored through evo-devo approaches.^121^

### Signalling pathways related to tooth agenesis

According to clinical reports, tooth agenesis-related pathogenic genes include Wnt10A, Wnt10B, Msx1, Pax9, TGFA and AXIN2, which are involved in multiple signalling pathways, such as Wnt/β-catenin, TGF-β/BMP and Eda/Edar/NF-κB.^122–126^ Therefore, even if many factors can prevent or interfere with the development of the tooth germ, such as genetic conditions, trauma, radiation and infectious diseases, studies have mostly focused on abnormalities in Wnt, Shh, Bmp and FGF signalling. To date, increasing mouse models have been constructed to understand the mechanism underlying tooth agenesis (Table 2).

#### Wnt signalling pathway

The Wnt signalling pathway is a major pathway responsible for human tooth agenesis.^127,128^ Different combinations of sequence variants in Wnt-related genes (such as *WNT10A*, *WNT10B*, *LRP6*, *AXIN2, KREMEN1*, etc.) lead to various patterns of missing teeth.^129^ Wnt10A mutations preferentially affect the permanent dentition instead of the deciduous dentition, indicating that the role of Wnt10A may vary between the development of deciduous and permanent teeth.^130^ Wnt10B mutations also tend to interfere with the development of permanent teeth, especially the lateral incisors.^131^

Investigations of mouse models also indicate that the Wnt signalling pathway plays a critical role in the occurrence of tooth agenesis. β-catenin is an important molecule in classical Wnt signalling and binds to TCF/LEF to stimulate the transcription of target genes of Wnt signalling.^132^ The loss of β-catenin in the epithelium prevents the tooth germ from developing into the bud stage.^41^ Additionally, after the conditional removal of β-catenin from the mesenchyme, the tooth bud fails to attain the cap stage.^133^ This phenomenon conforms with the observation that odontogenesis signals are transmitted from the epithelium to mesenchyme. Moreover, tooth development can be blocked by inhibiting Wnt signalling via decreasing the expression of the Wnt activator R-Spondin 3 or increasing the expression of the Wnt inhibitor, Dkkl.^89,134,135^ The ectopic expression of Wnt7b arrests tooth germ development, which is accompanied by decreased Shh expression.^99^

In addition, the model of mouse incisor germ demonstrated the importance of the Wnt signalling pathway in tooth agenesis, even if it belongs to hypselodont instead of brachydont (such as teeth of humans and molar of mice). The mouse incisor germ is at risk of developmental arrest owing to abnormality in the *Pitx2–Sox2–Lef1* axis (as mentioned in section 1.1). Conditional *Sox2* deletion in the dental epithelium arrests the development of incisors, which is related to absorption of the tooth germ caused by decreased stem cell proliferation and differentiation. This phenotype can be rescued via *Lef1* overexpression.^32^

#### SHH signalling pathway

Shh signalling appears to be specific to maxillary incisor development. The solitary median maxillary central incisor syndrome (SMMCI), a special kind of tooth agenesis in human, is associated with SHH pathway. Its pathogenicity gene includes *SHH, SIX3, TGIF1, DISP1, PTCH1, SMO,* etc..^136,137^ Besides, both *Gli2*^*−/−*^; *Gli3*^*+/-*^ and *Gas1*^*−/−*^; *Shh*^*+/*−^ mice have stunted maxillary incisors. And *Gli2*^*−/−*^; *Gli3*^*+/−*^ mice have smaller mandibular incisors and molars compared with wild type whereas *Gas1*^*−/−*^; *Shh*^*+/*−^ mice have varying combinations of midline-centred craniofacial deficiencies.^104,138^

#### BMP signalling pathway

BMP signalling, downstream of Wnt signalling, also contributes to early tooth development. Overexpression of epithelial Noggin (a BMP antagonist) can arrest tooth germ development,^139^ and knockout of *Bmpr1a* in the epithelium exhibits a similar phenotype.^140^ In addition to abnormalities in epithelial BMP signalling, knockout of some genes in the mesenchyme can prevent tooth germ development by affecting the epithelial–mesenchymal interaction. Knockout of BMP4 in the mesenchyme arrests the development of normal teeth and stagnates the formation of supernumerary teeth before the bud stage,^141^ and inactivation of mesenchymal Smad4 (TGF-β/BMP signalling) leads to the arrest of tooth germ development.^142^

Msx1 plays a critical role in the bud-to-cap transition by regulating the expression of Dkk2 and BMP4.^89,143^ Inactivation of Msx1 terminates tooth germ development at the bud stage,^144^ whereas deficiency of both Msx1 and Msx2 (*Msx1*^*−/−*^; *Msx2*^*−/−*^ double-knockout mice) arrests tooth development earlier at the placode stage.^145^ The arrested development of the maxillary molar germ in *Msx1*^*−/−*^ mice can be rescued by increasing the expression of Bmp4 or suppressing Dkk while inactivating Sfrp2 and Sfrp3.^107,146,147^

#### FGF signalling pathway

Failure of FGF signalling is identified as the main pathogenic mechanism in some tooth agenesis patients. Their mutated genes include *FGF3, FGF10, FGFR2*, and *FGFR3.*^148,149^ And according to the study on animal model, FGF signalling is important for the placode-to-bud transition, which is critical for the interaction between the epithelium and mesenchyme.^150,151^ In Fgfr2-deficient mice, tooth germ development is delayed and fails to progress beyond the bud stage.^152,153^ Moreover, the formation of both maxillary and mandibular molars is blocked before the bud stage in *Fgf3*^*−/−*^; *Fgf10*^*−/−*^ double-mutant mice.^154^

#### EDA signalling pathway

Mutations in the EDA signalling pathway (EDA, EDAR, EDARADD etc.) produce an ectodermal dysplasia phenotype that includes missing teeth in human.^155^ And the related genes may be associated with some specific sites of tooth agenesis. The hypodontia of the lower jaw incisors, second premolars and maxillary lateral incisors is associated with Eda and Edar mutations; and absence of molars, particularly the second molars of the lower jaw, is more likely to be associated with Pax9 mutations.^76,156–158^ Based on results from mice, Pax9 is involved with EDA signalling pathway during odontogenesis and its’ dosage has a direct impact on tooth development.^159–162^ Development of the tooth germ stagnates at the bud stage in *Pax9*^*lacZ/lacZ*^ mutant mice but progresses to the bell phase in *Pax9*^*neo/lacZ*^ mice.^163^

Besides studies on model animal and human mentioned above, tooth number abnormality of non-model animals or extinct animals provide new perspective to explore the pathogenetic mechanisms. An investigation from Megantereon supports that the occurrence of supernumerary tooth can be considered as evidence of atavism.^164^ In fact, the changes of tooth cusp in non-model animals or extinct animals gets more attention rather than tooth number abnormality.

## Connection between tooth number abnormalities and other human diseases

**Fig. 4:**
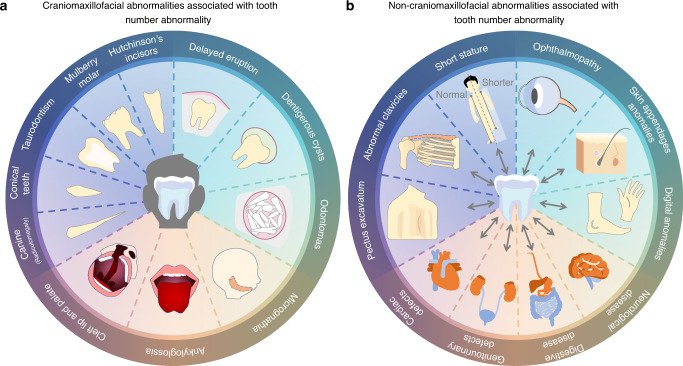
Human diseases connected with tooth number abnormalities. **a** Craniomaxillofacial abnormalities associated with tooth number abnormality. **b** Non-craniomaxillofacial abnormalities associated with tooth number abnormality

Numerous cases support a potential connection between tooth number abnormalities and other human diseases. In addition to common Craniomaxillofacial abnormalities, many systemic diseases such as skeletal system diseases, eye diseases, and nervous system diseases can also co-occur with tooth number abnormalities in patients with some syndromes (Fig. 4). To date, a mass of dentition-related syndromes have been identified (Table 3),^13,165^ and eight of those are more likely to be associated with the supernumerary tooth phenotype (Table 4).^166^ Therefore, a few syndromes can be diagnosed earlier based on the occurrence of supernumerary teeth. For example, in the case of FAP (OMIM 175100), abnormalities in its pathogenic gene, APC, can affect the activity of Wnt/β-catenin signalling to interfere with tooth development.^84,88^ Because dental abnormalities are usually detected earlier than gastrointestinal symptoms, supernumerary teeth and odontomas can serve as important diagnostic clues to FAP.^14,167,168^

**Table 3:** Phenotypes of supernumerary teeth-related syndromes

Syndrome	Oral symptoms	Other symptoms	Reference
Cleidocranial dysplasia	Delayed eruption of permanent teeth Hypoplastic maxilla Characteristic shapes of the ramus, condyle, and coronoid	Abnormal clavicles Patent sutures and fontanelles Short stature Pectus excavatum Sinus and middle ear infections	^206–209^
Familial adenomatous polyposis	Unerupted teeth Congenital teeth missing Dentigerous cysts Odontomas	Adenomas in the rectum and colon Osteomas Congenital hypertrophy of the retinal Pigment epithelium (CHRPE) Desmoid tumours	^84,210^
Nance-Horan syndrome	Notched incised edges (Hutchinson’s incisors) Mulberry molars Talon’s cusp Taurodontism	Congenital cataract Strabismus	^211–213^
Oculofaciocardiodental syndrome	Radiculomegaly facial anomalies cleft palate	Microphthalmia Congenital cataracts Cardiac and digital abnormalities neuropathy Muscle hypotonia Pituitary underdevelopment Brain atrophy Lipoma Childhood lymphoma	^214–216^
Opitz BBB/G syndrome	Cleft lip and palate Micrognathia Ankyloglossia High-arched palate	Hypertelorism Hypospadias Laryngo–tracheo–oesophageal abnormalities Neurological, anal, and cardiac defects Dysphagia Developmental delay	^217–219^
Robinow syndrome	Midline clefting of the lower lip Gum hypertrophy Dental crowding Ankyloglossia or “tongue tie” (bifid tongue)	Hypertelorism Nasal features (large nasal bridge, short upturned Nose, and anteverted nares) Midface hypoplasia Mesomelic limb shortening Brachydactyly Clinodactyly Micropenis Short stature Umbilical hernia	^220,221^
Rubinstein–Taybi syndrome 1 Rubinstein–Taybi syndrome 2	High palate	Moderate-to-severe intellectual disability Downslanted palpebral fissures Low-hanging columella Grimacing smile Talon cusps Short stature Obesity Eye anomalies	^222,223^
Trichorhinophalangeal syndrome type I	Thin upper lip Small jaw	Relative macrocephaly Sparse hair Bulbous nasal tip Protruding ears Prominent forehead Short hands and feet Bulbous pear-shaped nose Tented alae Long-extended philtrum Horizontal groove on the chin.	^224,225^

**Table 4:** Syndromes with supernumerary teeth phenotypes

Syndrome	OMIM	Gene	MOI
Cleidocranial dysplasia#	119600	RUNX2	AD
Familial adenomatous polyposis #	175100	APC	AD
Nance-Horan #	302350	NHS	XL
Oculofaciocardiodental syndrome #	300166	BCL6	XL*
Opitz BBB/G syndrome #	300000	MIDLINE 1	AD
Robinow #	180700	ROR2	AD
Rubinstein–Taybi syndrome 1# Rubinstein–Taybi syndrome 2#	180849 613684	CREBBP	AD
Trichorhinophalangeal syndrome type I #	190350	TRPS1	AD
Amelogenesis Imperfecta	204690	FAM20A	AR
Bloch-Sulzberger syndrome	308300	IKBKG	XL
Craniosynostosis	614188	IL11RA	AR
Crouzon syndrome	123500	FGFR2	AD
Ehlers-Danlos Type III	130020	COL3A1	–
Ehlers-Danlos Type IV	225400	PLOD	AR
Ellis–Van Creveld	225500	EVC, EVC2	AR
Fabry disease	301500	GLA	XL
Hallerman-Streiff	234100	Unknown	–
Noonan syndrome	163950	PTPN11	AD
Oral-facial-digital syndrome type I	311200	OFD1	XL
Rothmund–Thomson syndrome	268400	RECQL4	AR
SOX2 Anophthalmia syndrome	184429	SOX2	AD

*AD* Autosomal dominant, *AR* Autosomal recessive, *XL* X-linked

*Lethalon males

The potential relationship between tooth agenesis and tumours is noteworthy. Because of the intersection of tumorigenesis- and odontogenesis-associated signalling pathways, tooth agenesis may provide some information regarding cancer susceptibility.^12^ Wnt signalling-related genes, such as Wnt10A, Wnt10B, AXIN2, MESD, LRP6 and Dkk1, are pathogenic genes related to tooth agenesis.^169–171^ Patients with AXIN2 mutations are susceptible to colorectal cancer.^172^ Additionally, patients with hepatocellular carcinoma, prostate cancer, ovarian cancer and lung cancer may have AXIN2 mutations.^173^ In addition to Wnt signalling, the FGF signalling pathway (FGF3, FGF10 and FGFR2) is associated with cancer susceptibility.^174^ Moreover, Pax9 is not only a pathogenic gene related to tooth agenesis^175^ but also a transcription factor that can affect malignant transformation by maintaining the differentiation of squamous cells.^176^ However, large-scale epidemiological surveys with genetic mapping and follow-up studies on a molecular level are required for determining the correlation between tooth agenesis and cancer.^177,178^

Furthermore, stem cells from supernumerary tooth provide a new possibility to cure some diseases. Supernumerary tooth was an ideal source of postpartum stem cells^15^ as the feasibility of deriving stem cells from a supernumerary tooth has been demonstrated.^179^ Compared with normal dental pulp stem cells (DPSCs), human supernumerary teeth-derived stem cells (SNTSCs) may exhibit a higher proliferation rate and differentiation potential.^128^ In particular, their osteogenic differentiation can be enhanced by stimulating oncostatin M with the increased expression of BMP2, BMP4, BMP6 and RUNX2, which are genes involved in hard tissue repair.^180^ Stem cells derived from mesiodens also have a high proliferation rate and an immunophenotype similar to that of DPSCs.^181^ Owing to their immunomodulatory properties, SNTSCs can ameliorate the symptoms of systemic lupus erythematosus.^17^ Moreover, the viability and osteogenic differentiation of supernumerary teeth-derived apical papillary stem cells (SCAP-Ss) are better than those of DPSCs. SCAP-Ss are derived easily after birth and have considerable therapeutic efficacy for hepatic fibrosis.^16^

## Genetic intervention strategies for tooth number abnormalities

Tooth development depends on proper odontogenesis signalling. Abnormal signalling seems to be the primary cause of tooth number abnormalities. Therefore, these abnormalities can be treated by intervening with the signalling network, especially in tooth agenesis.^18^

Tooth agenesis caused by mutations in *Msx1* can be rescued via different genetic interventions. As mentioned before, the Bmp4–Msx1 pathway plays an important role during early tooth development, which can regulate the secreted Wnt antagonists, including Dkk2, Sfrp2 and Sostdc1.^143^ Additionally, Bmp4 promotes the transition of the arrested tooth germ (caused by *Msx1* mutations) from the bud stage to the bell stage in vitro.^147^ Genetic inactivation of *Sfrp2* and *Sfrp3* in combination with IIIC3a (a Dkk inhibitor) treatment can rescue the arrested *Msx1*^*−/−*^ tooth bud in vivo.^107^ Furthermore, understanding the mechanisms underlying the formation of supernumerary teeth provides novel insights into the treatment of tooth agenesis. Although *Msx1* is essential for normal tooth development, it is dispensable for the formation of supernumerary teeth in *K14-Cre*^8*Brn*^; *Apc*^*cko/cko*^ mice, which suggests that *Apc* escapes the Msx1–Bmp4 feedback loop to rescue tooth germ arrest and facilitates its development to more advanced stages.^88^ Sostdc1 (ectodin, Wise and USAG1) is another candidate protein for molecular targeted therapy of tooth agenesis. It was discovered by analysing unknown cDNAs of mice and humans and is located on the human chromosome 7p21.2.^182^ Sostdc1 has a strong restrictive effect on the spatial localisation of dental signals. Its inactivation leads to the formation of supernumerary teeth, and its excessive activation results in tooth agenesis.^91,93^ Animal studies have revealed that blocking USAG-1 function via USAG-1 knockout or using anti-USAG-1 antibodies can treat tooth agenesis caused by genetic factors such as Msx1, EDA1 and Runx2.^19,20^

Lef1 is required for tooth development and is related to tooth agenesis. It mediates the odontogenesis signalling network and is required for the relay of Wnt signalling to a cascade of FGF signalling during tooth morphogenesis.^38^ The loss of Lef1 causes tooth development arrest at the late bud stage and prevents the expression of multiple signalling molecules.^39,183^ These effects can be rescued by increasing the expression of *FGF4*, the downstream target of Lef1 and Wnt signalling.^183^ In addition, *Lef1* regulates the self-renewal ability of EpSCs based on the *Pitx2–Sox2–Lef1* interaction.^33,34^ The development of incisors is arrested in *Pitx2Cre*; *Sox2*^*F/F*^ mice owing to impaired proliferation of EpSCs and defective differentiation of dental epithelial cells. *Lef1* overexpression partially rescues tooth development arrest by forming a new EpSC compartment.^32^

The development of supernumerary tooth germ can be blocked by intervening with genes related to tooth agenesis. The formation of supernumerary incisors in *K14Cre*; *Fam20B*^*fl/fl*^ mice can be partially prevented by deleting *Sox2* from the dental epithelium, whereas it can be completely prevented by increasing the expression of *Dkk1.*^115^ The formation of supernumerary teeth relies on the hyperactivation of Wnt signalling. This hypothesis is also supported by the phenotype of *Gpr177*^*K14cre*^*/Osr2*^*−/−*^ mice. Proteins coded by *Gpr177* regulate Wnt sorting and secretion in mice. The formation of lingual supernumerary teeth in the molar region owing to the deficiency of Osr2 is arrested at the lamina/early bud stage because of epithelial inactivation of Gpr177.^54^

## Conclusion

This review elaborates on the key findings and recent progress of tooth number abnormality based on developmental biology, animal models, clinical diagnosis and treatment. Genes related to the occurrence of anomalies in tooth number have been described in detail, which may also contribute to the development of teeth regeneration in the future. Clinical information regarding the diagnosis and treatment of supernumerary teeth or tooth agenesis may help clinicians to diagnose and manage dentinogenetic abnormalities and other related systemic diseases. In addition, genetic intervention-based treatment approaches for abnormal teeth number have been summarised. Although these ideal treatments have been investigated only in animal studies, these studies provide a rationale for developing treatment strategies for tooth number abnormality in humans.
